# The Role of Chromosome Missegregation in Cancer Development: A Theoretical Approach Using Agent-Based Modelling

**DOI:** 10.1371/journal.pone.0072206

**Published:** 2013-08-26

**Authors:** Arturo Araujo, Buzz Baum, Peter Bentley

**Affiliations:** 1 Centre for Mathematics and Physics in the Life Sciences and Experimental Biology (CoMPLEX), University College London, London, United Kingdom; 2 MRC Laboratory for Molecular Cell Biology, University College London, London, United Kingdom; 3 Department of Computer Science, University College London, London, United Kingdom; Centrum Wiskunde & Informatica (CWI) & Netherlands Institute for Systems Biology, Netherlands

## Abstract

Many cancers are aneuploid. However, the precise role that chromosomal instability plays in the development of cancer and in the response of tumours to treatment is still hotly debated. Here, to explore this question from a theoretical standpoint we have developed an agent-based model of tissue homeostasis in which to test the likely effects of whole chromosome mis-segregation during cancer development. In stochastic simulations, chromosome mis-segregation events at cell division lead to the generation of a diverse population of aneuploid clones that over time exhibit hyperplastic growth. Significantly, the course of cancer evolution depends on genetic linkage, as the structure of chromosomes lost or gained through mis-segregation events and the level of genetic instability function in tandem to determine the trajectory of cancer evolution. As a result, simulated cancers differ in their level of genetic stability and in their growth rates. We used this system to investigate the consequences of these differences in tumour heterogeneity for anti-cancer therapies based on surgery and anti-mitotic drugs that selectively target proliferating cells. As expected, simulated treatments induce a transient delay in tumour growth, and reveal a significant difference in the efficacy of different therapy regimes in treating genetically stable and unstable tumours. These data support clinical observations in which a poor prognosis is correlated with a high level of chromosome mis-segregation. However, stochastic simulations run in parallel also exhibit a wide range of behaviours, and the response of individual simulations (equivalent to single tumours) to anti-cancer therapy prove extremely variable. The model therefore highlights the difficulties of predicting the outcome of a given anti-cancer treatment, even in cases in which it is possible to determine the genotype of the entire set of cells within the developing tumour.

## Introduction

Cells with a wide range of structural and numerical defects in chromosomes are found in many types of cancers. Whether these changes contribute directly to the evolution of cancer or are just a by-product of carcinogenesis itself, however, is a question that has puzzled cancer researchers for more than a century. Although there is strong experimental evidence for changes in chromosomal copy number (aneuploidy) and chromosome mis-segregation playing a central role in the way cancer evolves [2], no organizing principles or clear evolutionary pathways have been established. Therefore, an alternative approach is to study the problem from a theoretical viewpoint, using simple computational models of cell behaviour and cell-cell interactions to study homeostasis, its dysregulation during cancer progression and its response to treatment [3].

Computational modelling has recently become a practical approach for the study of such emergent behaviours and complex phenomenon [4]. Agent-based models have been used with success to model the complexity found in ecological [5], economical [6] and cancer systems [7], [8]. In complex systems, global behaviour emerges from the interactions of the individual components, and cannot always be inferred from an analysis of the individual components in isolation [9]. Instead, however, agent-based models can be used to determine the effects of interactions between individual components on the behaviour of the system as a whole [10]. One of the main advantages offered by agent-based modelling over equation-based modelling techniques is the ability to study the emergent behaviour that arises from defined interactions between elements of a complex system [11]. Because cancers are made up a large number of cells of diverse genotypes that interact without centralised control, agent-based modelling may help capture the essence of the system from the behaviour of individual cells. Inspired by this type of computationally tractable model, we have developed a framework with which to analyse the role of chromosomal instability in cancer progression, and to investigate the impact of chromosome mis-segregation in cancer treatments. In silico experiments were then carried out to simulate the interaction between chromosome mis-segregation and cancer treatments; including abstractions of surgery, the physical removal of tumour mass, chemotherapy, a treatment where over-proliferating cells are targeted and killed; and a combination of these two treatments. It is clear from simulations that cancers with an unstable complement of chromosomes have a worse overall prognosis. Moreover, the two types of therapy work in distinct ways enabling them to be combined to further delay the course of cancer progression. Finally, the analysis makes clear the difficulties of predicting the course of any one cancer or its response to therapeutic intervention.

## Results and Discussion

### The Model

**Figure 1:**
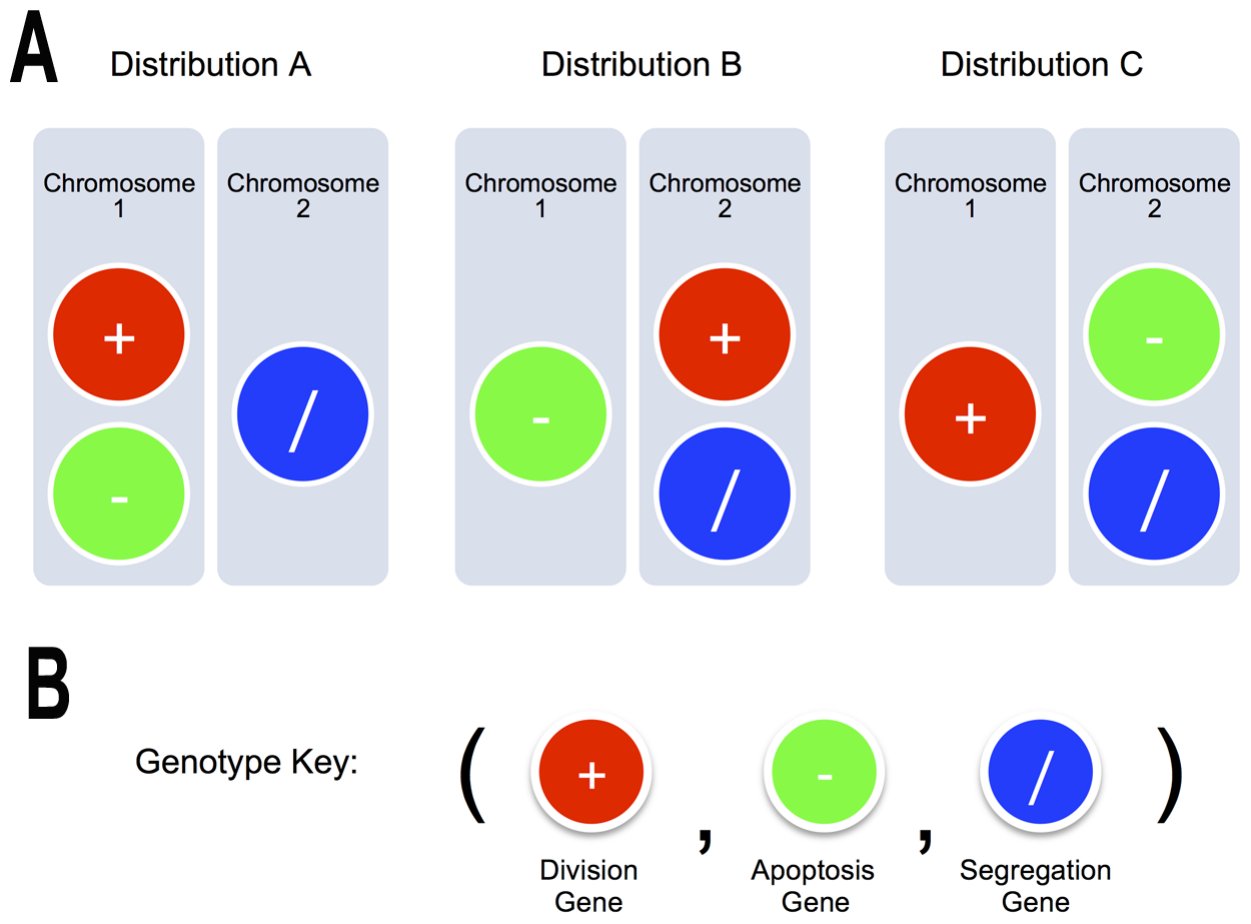
Genotype configurations and Gene Key. **A.** The different Gene Abstractions were placed into chromosomes in three different configurations. This led to different kinds of linkages between the Genes. **B.** For the notation of different genotypes, we have used the following key: (Number of Division Genes, Number of Death Genes, Number of Segregation Genes). The initial Genotype in every simulation is a diploid genome: (2,2,2). To better understand the proportions of the genes in a given phenotype, we have used the RGB model to represent the number of division genes as red, the number of death genes as green and the number of segregation genes as blue See Methods, Genotype Key).

To address whether chromosome missegregation plays an important role in the development and progression of a cancer we developed a simple model of tissue homeostasis in which to study cancer evolution. To focus our analysis on this poorly understood phenomenon we chose to disregard other types of mutations (such as substitutions, insertions, deletions, and chromosome translocations). For this, individual cells were modelled, each equipped with a genetically defined genome, as agents in a computational simulation (see Methods). We then represent the tissue as a linear array of individual cells, where daughter cells are spatially introduced adjacent to the mother cell of origin. The simulated tissue initially exhibits homeostatic behaviour, as the result of balanced rates of cell proliferation and cell death. These behaviours were modelled as stochastic processes that are regulated at a genetic level, based upon the properties of known proto-oncogenes and tumour suppressor genes [12]. While in real biological systems many features of cell biology are polygenic, we made the simplifying assumption that a single gene dominates in the regulation a specific behaviour, and that the impact of each gene is proportional to the number of copies of a given gene found in the genome of each cell, as suggested by recent studies on the effects of differences in chromosome number on gene expression in biological systems [13], [14]. This simplification is a necessity while the human genetic regulatory network remains unknown. In addition, it is key to understanding the effect of missegregation events that affect chromosomes containing key genes such as p53, Ras and pRb [12]. Thus, while reality is much more complicated, we anticipate that it will be possible in the future to apply the insights obtained by addressing this fundamental problem in an abstract way to human cancer. Having established this model system, we then introduced a gene abstraction that regulates fidelity during cell division, which enable us to test the role of evolving chromosomal instability in cancer development and treatment. In this way, we can isolate the effects of chromosome instability, tumour suppressor and oncogene activity and genetic linkage on cancer progression (see Figure 1 A).

Each cell in the system has a simulated genome composed of three kinds of genes. **Apoptosis regulatory genes** are an abstraction of tumour suppressor genes such as *p53* [15] that regulate cell death, and enable us to model the fact that tissue crowding leads to a corresponding increase in the rate of delamination and cell death within an epithelium to maintain homeostasis [16] [17]. To balance cellular death, **cell division regulatory genes** provide an abstraction of proto-oncogenes such as Ras [18], Myc [19] and p110 PI3K [20] and act to promote cell growth and cell cycle progression. Again the action of these genes is sensitive to the “homeostatic capacity” of the tissue in order to model the process know as contact inhibition that limits cell proliferation in crowded tissues [17]. Thus, in combination these controls ensure that if the number of cells exceeds the homeostatic limit, proliferation is inhibited and the probability of cell death increased, maintaining a constant population of cells close to the homeostatic capacity of the simulated tissue.

In addition, the model contains a finite rate of chromosome mis-segregation during cell division, which generates variation amongst the cell population. This level of genetic variation depends on the action of **chromosome segregation regulatory genes**, which model genes controlling the fidelity of cell division such as BUB1 [21] and MAD2 [22] that reduce the likelihood of chromosome mis-segregation at cell division. In the initial population of cells, each cell has two sets of identical chromosomes (a diploid genome) and 2 copies of the chromosome segregation gene. When dividing, the genome of each cell is duplicated and the two sets of chromosomes are then segregated into two daughter cells. It is during this stage that chromosome mis-segregation events can occur, resulting in asymmetric cell division: one daughter cell with an extra chromosome, and one lacking the same chromosome.

### Simulating Chromosome Missegregation

Because the exact role, location and linkage of the key genes regulating cell growth, death and chromosome segregation in real human chromosomes remains unknown [23], here we have also explored how differences in the distribution of genes on chromosomes affects the evolution of the system as a whole. To do this, we placed the abstracted genes in three different chromosomal configurations (Figure 1 A). These are distribution A, where apoptosis regulatory genes and cell-division regulatory genes are “linked” in the same chromosome; distribution B, where cell-division regulatory genes and chromosome segregation regulatory genes lie on the same chromosome; and Distribution C where genes regulating apoptosis and chromosome segregation are genetically linked. At the start of simulations each cell was then modelled as a diploid, containing two copies of each chromosome (Figure 1 A).

**Figure 2:**
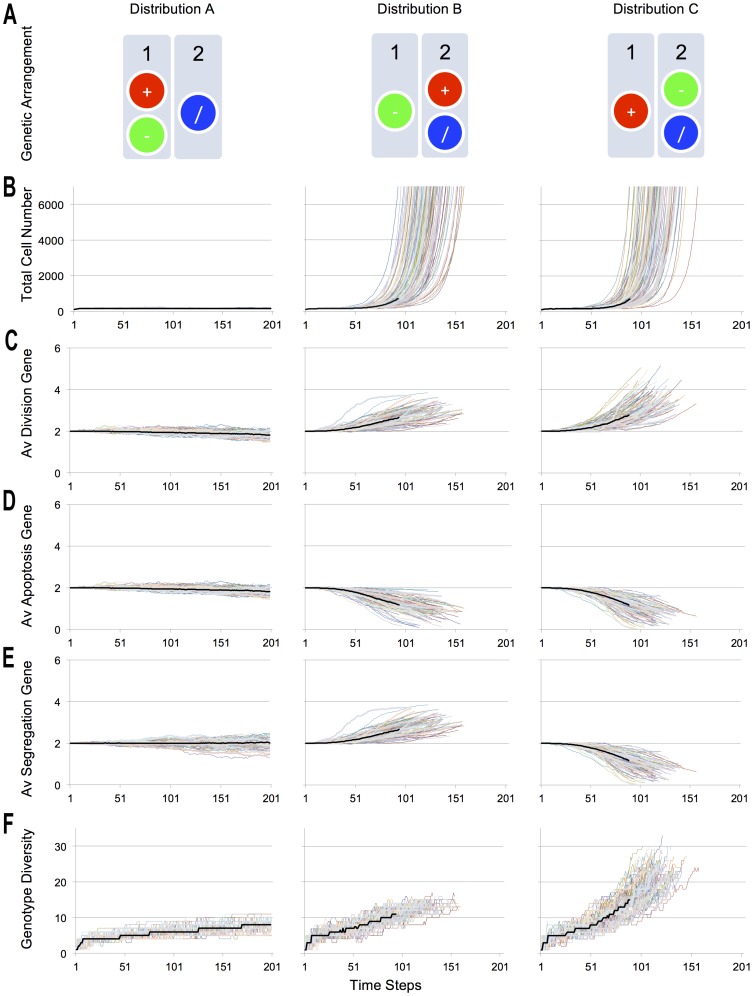
Analysis of simulations. **A.** The three genetic arrangements, in simulated diploid chromosomes. Key measurements of each configuration are represented in Broom Diagrams. **B.** Aspects of each simulation, from total number of cells to genetic diversity are represented as line of different colour, with the median as a thick, black line (calculated until one of the simulations came to an end). The behaviour observed for Gene Configuration A is a homeostatic one. Configurations B and C displayed an over-proliferative behaviour. This is due to the genetic up and down regulation reflected by the change in the average number of key genes across time. **C.** The average number of Division Genes. **D.** The average number of Apoptosis Genes. **E.** The average number of Segregation Genes. **F.** The genetic diversity, liked to the number of Segregation genes, had a profound effect on the Genotypic Diversity, being greatest in Configuration C. Colours are purely used to distinguish runs and do not denote genetic distribution.

The evolutionary dynamics in our model are then determined by the gene expression of the individual cells and the global behaviour that emerges through cell death, proliferation and mis-segregation over time. Focusing on the genotypes that emerge throughout the simulation, we denote the initial state as (2, 2, 2): corresponding to 2 functional copies of each gene (Division, Apoptosis and Segregation, respectively as seen in Figure 1 B). Cancer-like growth will ensue if the number of oncogenes increases and/or if all tumour suppressors are lost. Exploring the three different gene distributions, 100 simulations were performed for each configuration (Figure 2 A). Because instances of cell division, birth and cell death are expected to be stochastic in nature, and have been modelled as such, the behaviour of the system is highly variable. Nevertheless, consistent trends can be observed as illustrated in Figure 2 B.

First, Gene Distribution A resulted in homeostatic behaviour, in which the system as a whole responds to fluctuations in cell number to maintain the total number of cells close to that of the carrying capacity of the tissue (200 cells). As expected, the plot of the total number of cells across the simulations of Distribution A revealed increasing variability in the genetic make-up of individual cells over time as the result of chromosome mis-segregation induced genetic drift; similar to that which might be seen in an ageing homeostatic tissue. Although this variation makes the statistical analysis challenging, an invariant behaviour can be observed for each configuration; best visualized by broom plots in Figure 2 B. In this case, because the abstracted genes that model the role of oncogenes and tumour suppressor genes were coupled by being situated on the same chromosome, the balance between death and division was maintained despite the generation of new genotypes emerged through chromosome mis-segregation events. Significantly, some of the more successful genotypes naturally acquired more resistance against chromosome mis-segregation, through the acquisition of an extra copy of the chromosome segregation regulatory gene (genotype state (2,2,3)), as seen in Figure 2 E. This kind of stable aneuploid karyotype is found in normal homeostatic tissues [24].

**Figure 3:**
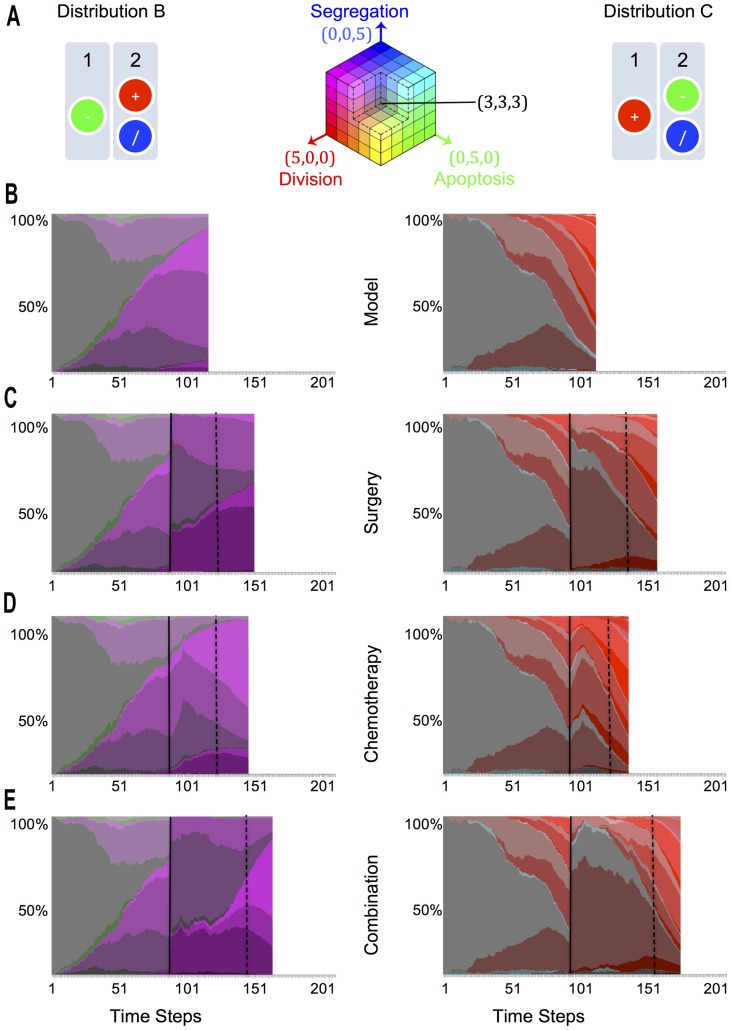
Genotype diversity. **A.** The two over-proliferative genetic arrangements, in simulated diploid chromosomes, and the RGB key in the middle. We have used the RGB colour model to visually describe the different genotypes that evolve in the system by normalizing the maximum observed Genotype State (See Methods, RGB Key). We have assigned a colour to each of the abstracted genes: Red for division, green for death and blue for segregation. By comparing via an RGB system the colours assigned to a given genotype, we are able to tell visually the proportions in which the genes are distributed, with intensity values corresponding to the number of genes: (0,0,0) being black, the initial genotype (2, 2, 2) being dark grey and the maximum observed genotype (5, 5, 5) being white. **B.** Representative Marble Diagram for a simulation with the Model. These diagrams display the stacked percentage of Genetic Diversity across time for a representative simulation of Gene Configurations B and C across different scenarios. The beginning of therapies (when reaching 1000 cells) are marked with a black vertical line, while relapse times (when reaching again 1000 cells) are marked using a dashed line. **C.** Representative Marble Diagram for a Simulation of Surgery. **D.** Representative Marble Diagram for a Simulation of Chemotherapy. **E.** Representative Marble Diagram of a therapy combination of Surgery followed by Chemotherapy.

For Gene Distribution B, the gradual accumulation of chromosome mis-segregation events leads to a breakdown in homeostatic behaviour, giving rise to uncontrolled proliferation (Figure 2 B). Once this occurred, total cell number increased exponentially, reaching the values of the order of thousands in a very short period of time. This kind of over-proliferative behaviour was consistent across simulations. An analysis of the emergent genotypes evolved through Gene Distribution B, as seen in Figure 3 B, revealed that aneuploid genotypes such as (3,2,3) and (2,1,2) take over the population. From these aneuploid genotypes, initially only slightly different to the original one, the population branches out to generate more malignant genetically distinct variants such as (3,1,3) and (2,0,2). Different kinds of successful (and less successful) genotypes are gradually evolved. Successful genotypes have the qualities of being apoptosis-resistant (low number of apoptosis genes, as seen on Figure 2 D) and over-proliferative (increased number of division genes, as seen on Figure 2 C). In this distribution, however, because the genes that regulate division are coupled to those that regulate fidelity during segregation (Figure 2 E), there is a brake applied to the subsequent generation of aneuploid genotypes with increased division rates. As a result, this population of aneuploid cells remained relatively homogeneous once cells had acquired the key genetic anomalies driving deregulated tumour growth (Figure 2 F). This kind of evolution observed across experiments suggests a possible pathway for oncogenesis that is associated with stable aneuploidy [24]. Diseases such as leukaemia, lymphomas and some mesenchymal tumours that exhibit specific abnormalities may follow a similar path [25].

Simulations of Gene Distribution C displayed over-proliferative behaviour, similar to that of Gene Distribution B (Figure 2 B). On a closer inspection, however, significant differences in the dynamics of cancer evolution were observed (Figure 3 B). Because the genes that regulate death are genetically linked to those that regulate segregation in Gene Distribution C (Figure 2 D and Figure 2 E), cancer evolution was accompanied by an increase in genotypic diversity as the drive to lose apoptosis regulators leads to a concomitant deregulation of chromosome segregation (Figure 2 F), as in genotype (3,1,1) and then genotype (3,0,0). This in turn drives to the emergence of ever more aggressive clones (4,0,0), (5,0,0) and (6,0,0), which corresponds to a 3-fold increase in the rate of cell proliferation (Figure 3 B). This serves as a model for the emergence of heterogeneous tumours, like those seen in clinical settings, for example during the neoplastic progression characteristic of epithelial tumours [26] [27]. These simulations for Distribution B and C show how chromosome mis-segregation events can drive tumour evolution by breaking the regulatory balance that maintains normal tissue homeostasis.

To test the effects of leaving genes unlinked, a fourth genetic distribution was investigated by modifying the model to accommodate a third chromosome. This system exhibited all three behaviours obtained previously in stochastic simulations: prolonged homeostasis (as in Distribution A), unregulated growth driven by loss of tumour suppressors (Distribution B) or by oncogene activation (Distribution C), We also observed three kinds of chromosome segregation event: up-regulated (Distribution B), down-regulated (Distribution C) and neutral. This control experiment shows how linkage between genes serves to limit the common evolutionary paths exhibited by the system.

### Chromosome Missegregation in Cancer Therapies

In patients, tumours composed of cells that are chromosomally unstable have been associated with a poor prognosis [28]. We therefore used Gene Distributions B and C (Figure 3 A) to determine the relative efficacy of different treatment strategies in dealing with tumour evolution under conditions of low and high levels of genome instability. We considered tumour detection would occur when the population reached 1000 cells. By the same token, we considered that the tumour had relapsed when it again reached the 1000 cell mark after treatment (marked as vertical lines in Figure 3). Using these measures, we modelled the outcome of different treatments on single tumours (or patients), so that we could directly compare the outcomes in each case, despite the expected variability in the course of tumour growth between different simulations (tumours/patients). Data for a representative experiment for each simulation are shown in Figure 3.

Scenario i: Surgical Treatment. The simulation of tumour removal was implemented by retaining the first 100 connected cells in the linked list and removing the rest of the connected component of 900 cells in a single time-step. Since the tumour rapidly emerged from a homeostatic population of 200 cells, the vast majority of these represent cells related to cells in the tumour. Scenario ii: Chemotherapy: To simulate the effects of chemotherapy, we implemented an algorithm that killed all the cells that attempted cell division in the nine consecutive time steps following tumour detection. Scenario iii: Combination Therapy. As in common in the clinic we combined therapies by implementing surgery followed by nine rounds of chemotherapy.

Surgery was modelled to mirror the clinical intervention. Thus, it was implemented when the population of cells has broken through the homeostatic limit of 200 cells, and grown to reach 1000 cells. At this point, the population is made up of descendants of many of the cells present in the initial population used to seed the simulation, but is dominated by a small number of related but genetically heterogeneous aggressive cell clones, as in human cancers [29]. The population also includes cells poised in a pre-cancerous state that are the product of a process analogous to field cancerization [30] which occurs as cells compete for space during the course of simulations. These pre-cancerous cells will be likely be related by lineage to the aggressive sub-clones that constitute the bulk of the tumour. At this point, 90% of the population were removed (Scenario i). To implement this, “adjacent” cells were removed from the cell list to mimic surgical removal of the tumour bulk. It is important to note that these cells tend to be related by lineage as the result of cell division, as do the 10% of cells that remain.

**Figure 4:**
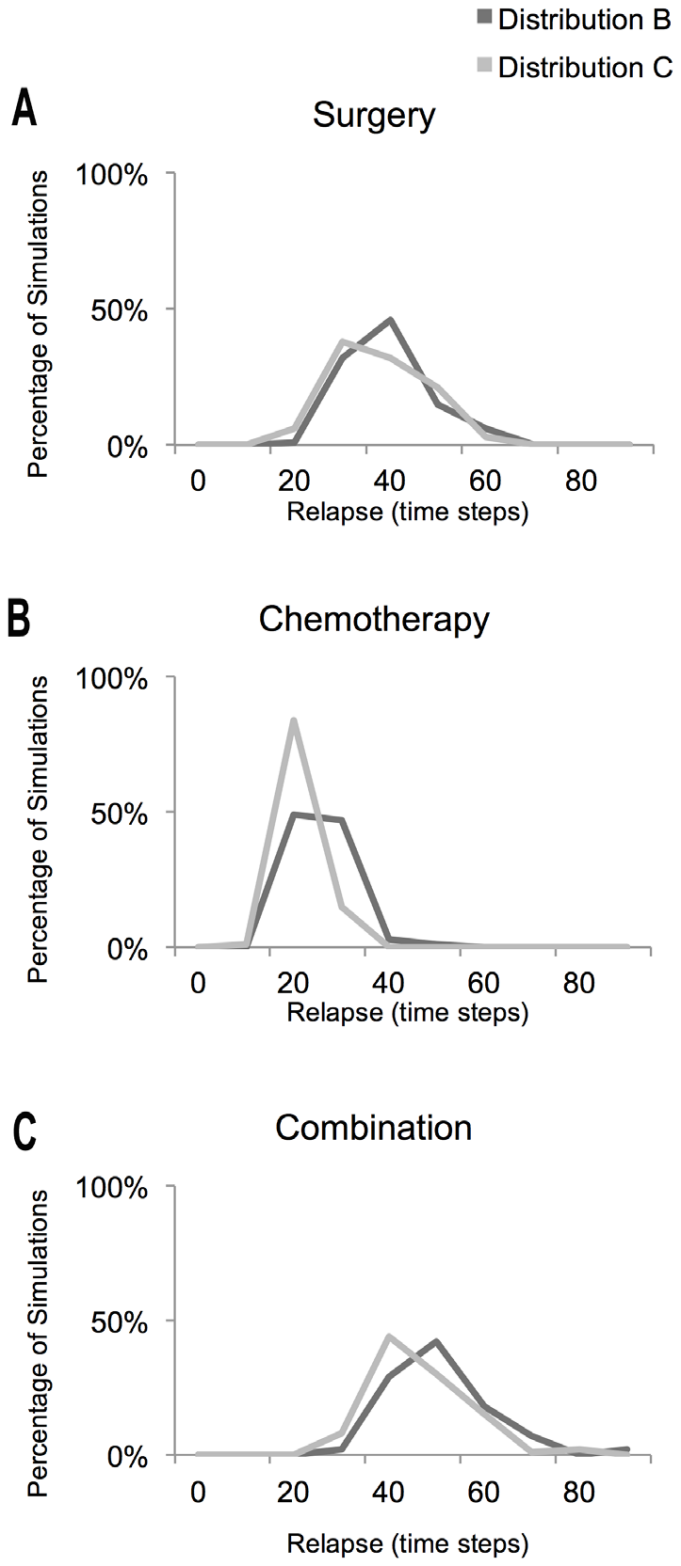
Distribution of the response to treatments under different scenarios. The histograms correspond to a measure of the distribution of the relapse times (the time it took each simulation to grow back to 1000 cells after treatment) for 100 simulations of each gene configuration under three different therapy scenarios: **A.** Surgery Scenario, **B.** Chemotherapy Scenario and **C.** Combination of both treatments (Surgery followed by Chemotherapy).

When we then examined the recovery following therapy, results proved highly variable and depended on the nature of the cells that survived (Figure 3 C and Figure 4 A). Though the actual evolutionary pathways exhibit a high degree of variation across simulations, a representative experiment for each gene distribution captured qualitatively the kind of evolutionary pathway that most of the simulations followed, as shown in Figure 3 C. After surgery an average of 105 cells were left (std. 4.50) for distribution B and 106 cells (std. 5.13) for distribution C. However, over 100 simulations the prognosis was significantly better (p = 0.0499) for tumours with Gene Distribution B, which exhibit relatively low levels of chromosome mis-segregation (relapse time was an average of 35.22 time steps and a standard deviation of 8.33), compared to those with Gene Distribution C and high levels of chromosome mis-segregation (with an average of 32.84 and a standard deviation of 8.70), as seen in Figure 4 A. This behaviour was due in part to the greater likelihood of a relatively normal population of cells remaining after surgery from a population with low genetic heterogeneity in comparison to that from a highly heterogeneous population. Simulation to simulation variability in the path to relapse was determined in part by the kind of genetic aberrations present in the population remaining following surgery. Thus, remaining cells that had suffered a loss of tumour suppressors would not over-proliferate until they underwent additional mis-segregation events, delaying the period of time until relapse. In contrast, for simulations in which over-proliferative genotypes are the first to emerge, a subset of cells remaining after surgery quickly re-grow to break through the homeostatic limit (inhibiting the growth of normal neighbouring cells through competition for space) to form a tumour. Thus, the relapse time in simulations is determined, primarily, by the oncogenic load, which is higher in the chromosomally unstable populations.

**Figure 5:**
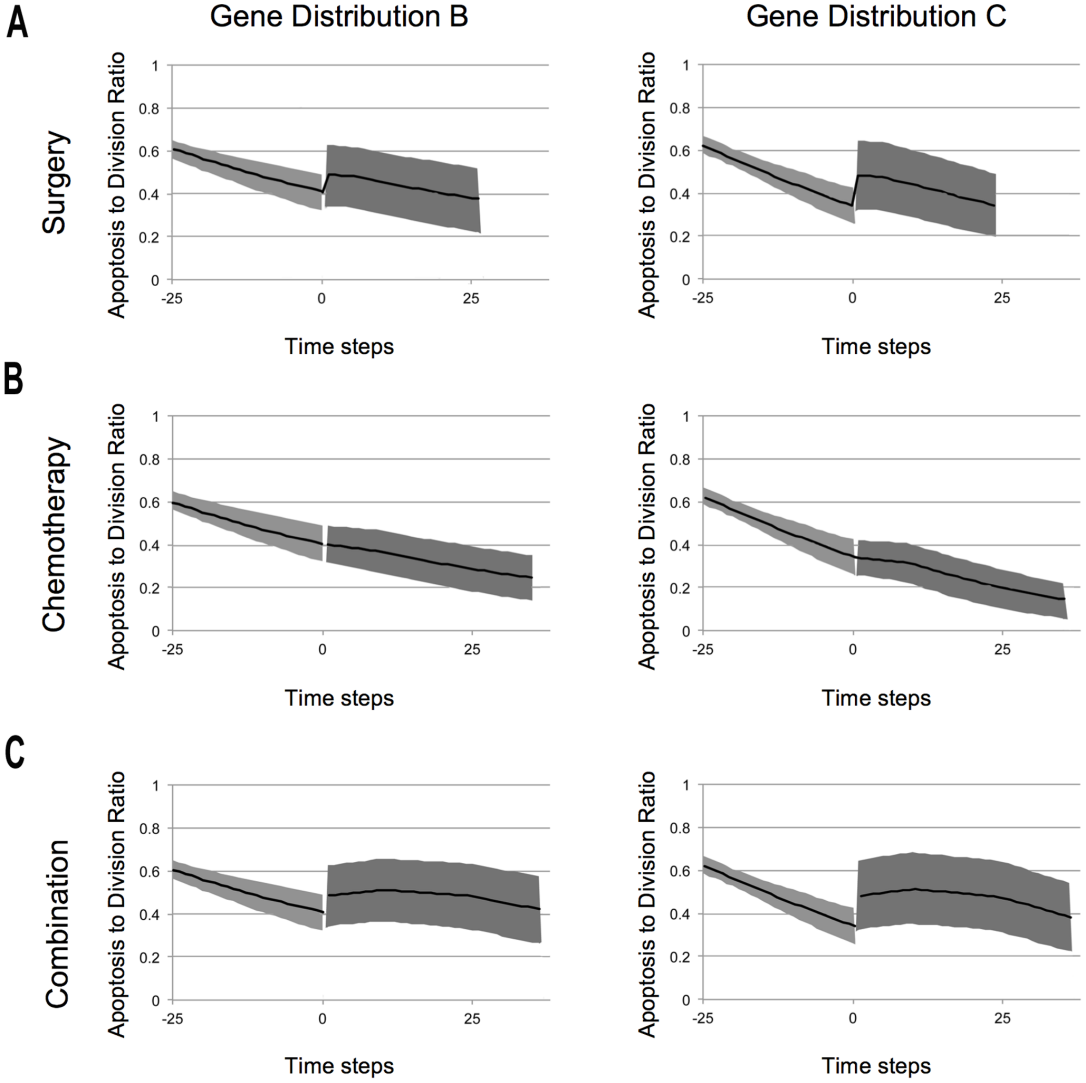
The average ratio of apoptosis to division genes. These graphs show the tendency of reducing the number of apoptosis genes and increasing the number of division genes with respect to time across different scenarios: **A.** Surgery Scenario, **B.** Chemotherapy Scenario and **C.** Combination of both treatments (Surgery followed by Chemotherapy). The dark line is the median of the samples and the shadowed area represents the variance. Interventions were carried out at time step zero. The reported slopes were measured taking into account 25 time steps after each therapy.

Next we explored the role of genetic linkage in the course of tumour relapse following surgery. For this analysis, as a measure of the types of lesion driving tumour formation and relapse, we compared the ratio of the average number of Apoptosis Genes to the average number of Division Genes in simulations (shown in Figure 5 A). When this was analysed in the 25 time steps after surgery, it was clear that Gene Distribution C has a reproducibly higher rate of loss of Tumour Suppression and Oncogene acquisition than Distribution B. This can be seen most clearly by comparing changes in the rate of the ratio of the average number of Apoptosis Genes to the average number of Division Genes following treatment (Figure 5 A), which has an near linear slope of −0.0067 (std. 0.0037) for Distribution C, which is significantly steeper (p = 0.005E-1) than the average slope for Distribution B (slope −0.0049, std. 0.0030). This reflects the greater generation of more malignant novel genotypes in type C simulations, where chromosomal instability is high, compared to simulations for Distribution B, where aneuploidy is relatively stable. This in turn correlates with a worse prognosis for the genetically unstable tumours. Thus, in our simulations, surgery acts as a hit-or-miss therapy because it leaves cells that are related to each other.

Having carried out an analysis of the effects of surgery, we next simulated chemotherapy in the model (Scenario ii). Chemotherapy was implemented in consecutive rounds, as done in the clinic using a treatment such as taxanes, to specifically target dividing cells [31]. This therapy tends to remove cells of the tumour that have deregulated division, but also targets cells in the pre-cancerous population that have deregulated proliferation and normal cells that happen to divide. After chemotherapy, an average of 226.17 cells (std. 53.12) were left for Distribution B and 231.88 (std. 50.06) cells for Distribution C. These cell numbers reflect the mechanism by which chemotherapy acts: killing an average of 15.76% of the population of Configuration B (std. 0.47), and 16.3% of the population of Configuration C (std. 0.70) at each time step. In this way the course of treatment drives an exponential decrease in the number of cells killed.

When examining the effect of genetic linkage on the recovery following chemotherapy we found that the relapse time was again faster for cell populations with Gene Distribution C. Thus, Gene Distribution B relapsed on average at 21.95 time steps (std. 4.89), while tumours recurred in Gene Distribution C at an average of 18.30 time steps (std. 3.42), as seen in Figure 4 B. Again, this significant difference (p = 0.003E-5) in relapse time could be attributed to differences in genetic diversity between the two populations at the time of treatment. Moreover, when we measured the rate of acquisition of new variants that have increased oncogenic load and a reduced number of tumour suppressor genes (the ratio of the average number of Apoptosis Genes to the average number of Division Genes) there was a marked and significant difference (p = 0.004E-7) between simulations over 25 time steps after chemotherapy - an average slope of −0.0048 (std. 0.0016) for Distribution B, and −0.0068, (std. 0.0019) for Distribution C (as seen in Figure 5 B). This reflects the presence of higher numbers of cells poised in a pre-cancerous state following treatment in Distribution C.

Finally, a combination of the two therapies (Scenario iii) yielded an overall better prognosis for the two gene distributions than surgery or chemotherapy alone. After this combined therapy there were on average 36.09 (std. 8.56) cells left for Distribution B and 36.29 (std. 7.99) cells for Distribution C. Again, the results indicate that Gene Distribution B still has a significantly better prognosis (p = 0.008) than Gene Distribution C: Gene Distribution B had an average relapse of 46.55 (std. 10.06), while Gene Distribution C had an average relapse of 43.09 (std. 9.44). These results can be compared across scenarios in the form of histograms in Figure 4 C. Again, the overall impact of genetic linkage on the evolution of the tumour after treatment can be most easily visualized by comparing the average slope of the ratio between Apoptosis and Division Genes (Figure 5 C). When we considered the 25 time steps after therapy, this shifted significantly (p = 0.005E-2): −0.0036 (std. 0.0025) for Distribution B and −0.0052 (std. 0.0034) for Distribution C.

## Conclusions

Tumours have been recognised as aneuploid for over a century [32]. In addition, recent genomic sequencing studies have revealed enormous heterogeneity within single tumours, the role and consequences [33]. Nevertheless, the role of chromosome mis-segregation in cancer development is still debated, and experiments testing the effects of perturbing rates of chromosome segregation on tumour formation in mice have yielded contradictory results [34]. Therefore, to better understand the roles of chromosomal instability in the evolution of tumours, we decided to take a theoretical approach and developed an agent-based model of whole chromosome mis-segregation during cell division, in which to determine how chromosome mis-segregation might contribute to cancer initiation.

For this purpose, we focused on modelling individual cells and their genomes in a homeostatic tissue whose behaviour is determined by a balance of cell death and cell proliferation within the context of a constrained environment. When events of chromosome mis-segregation are introduced, however, the dynamics of the system change in such a way that new, interesting complex behaviours emerge, which can be used to shed some light on the basic principles of aneuploidy in tumourigenesis. In simulations, chromosome mis-segregation events generate novel genotypes that promote unregulated cellular proliferation and impair cellular death, driving cancer development. Importantly, this analysis also revealed that the location of these genes across chromosomes plays a key role in determining the system’s behaviour and in shaping the genetic structure of the tumour population [35]. This is driven by the fact that the copy number of genes that regulate the fidelity of chromosome segregation can alter as the result of the mis-segregation of their host chromosome at cell division. As a consequence, differences in the rates of mis-segregation evolve during the course of tumour development in a way that depends on genetic linkage. So, for example, in the absence of direct selection for chromosomes based upon the presence of genes promoting or inhibiting cell proliferation (Gene Distribution A), we observed a reproducible increase in the number of clones with a decreased rate of chromosome mis-segregation.

In our model we observe two distinct pathways for evolution towards oncogenesis that have a direct impact on the tumour’s response to treatments. In the first case, dominant proliferating clones within the tumour exhibit a relatively stable state of aneuploidy driven by the acquisition of genes that ensure the fidelity of chromosome segregation along with division genes that encourage proliferation. In the second, selection for the loss of the chromosome segregation regulatory genes together with the loss of tumour suppressors results in tumours that continually generate increasing levels of heterogeneity and ever-more malignant subclones. This latter pathway exhibited a more rapid expansion, suggesting that chromosomally unstable tumours are inherently more aggressive.

In this analysis we also explored the effects of 3 different types of simulated treatment in each case: surgery, chemotherapy and combination therapy. When comparing surgery and chemotherapy, it is important to note that the way in which the two therapies are implemented has important implications for interpreting the relapse data. First, when studying the effects of surgery, it was necessary to compare the genetic make up of cells in the simulation immediately before and after surgery. To do so, we compared the genetic make up of the 1000 cells in the time-step prior to the intervention, with the average of 100 cells in the time step following the intervention. The abrupt change in cell numbers generates a shift in make-up of the population, which leads to a marked increase in apparent population heterogeneity immediately following treatment. By contrast, when the chemotherapy treatment is implemented in a population reaching 1000 cells, the intervention leads to a variable decrease in population size with each time step that depends on the proliferation rate. On average this yielded a 16% decrease in the population size (840, 706, 593, 498, 418, 351, 295, 247 and 208 in the last time step of the intervention). This caused a smoother transition that depends on the precise impact of the treatment on the population.

These differences are reflected in the larger variation across simulations in the response to surgery compared to chemotherapy (Figure 5), which translates into chemotherapy being a consistent therapy, while surgery is a “hit or miss” therapy that can on occasion cure the tumour. Thus, although surgery appeared to have a better overall prognosis than chemotherapy in the simulations, there is a lag time required before the population following surgery recovers reaches levels seen following chemotherapy. Thus, it took 9 time steps for Configuration B to recover to yield an average of 210 cells (std. 33.08) and 8 time steps for Configuration C to reach on average of 205 cells (std. 42.64). When this difference is taking into account, both therapies are seen to yield a similar overall prognosis, which enables the differences in the patterns of recovery in the two cases to be usefully compared using this implementation (Figure 5 A and Figure 5 B).

Nevertheless, clear differences in the trajectory of relapse were seen following surgery and chemotherapy. A comparison between the ratio of the average number of Apoptosis Genes to the average number of Division Genes following surgery (Figure 5 A) and following chemotherapy (Figure 5 B) helps to reveal the key differences. In the first case, the mean number of apoptosis regulatory genes is, averaged over many simulations, increased towards diploidy following surgery. At the same time as the average number of division regulatory genes is decreased towards diploidy across simulations. This sharp increase in the average number of apoptosis genes and decrease in the average number of division genes gives rise to a sudden change in the slope of their ratio as depicted in Figure 5 A. It is important to note that while in some cases the recovery to diploidy was almost total, in many others the recovery was less pronounced or even counter-productive. Although on average a dramatic shift towards diploidy of a given chromosome can be observed, the spread of the results point to a large number of cases where surgery leads to the total loss of tumour suppression (with intact diploid division genes), an accelerated gain in oncogenes (with normal levels of tumour suppression) or a lethal combination of both. This leads to variability in the outcomes of surgery that depend on the limits of the cell population removed [29], and the extent of field cancerization in the tissue [30]. These subtle but complex dynamics in surgery are best observed on a case-by-case examination of the evolved genotypes, as those shown in Figure 3 B and Figure 3 C.

Under the chemotherapy scenario, the treatment also leads to an average reduction in the oncogenic load, as the result of the selective killing of dividing cells. This therapy, does not however offer relief from the steady loss of genes regulating apoptosis. As a result, the ratio of apoptosis genes and division genes remained, on average, nearly constant under these conditions, as can be seen by a slight change in the slope of their ratios after chemotherapy (Figure 5 B). It seems intuitively likely that chemotherapy, by selectively targeting actively proliferating cells, would prove a more effective treatment than surgery. However, our model shows that the result of treatment is complicated by the fact that there are two kinds of cells that underlie relapse. There are cells that have lost tumour suppression and cells that have acquired oncogenes. While surgery acts against both types of cell, chemotherapy cannot target cells that are no longer subject to apoptosis-mediated tumour suppression but which divide slowly. As a result of this bias, the recovery following treatment is characterised by different trajectories in the ratio of Apoptosis to Division Genes in the two cases. This unexpected result highlights the importance of these kinds of abstract models as aids to understanding the likely path of tumour evolution.

It is also possible to use these simulations to examine the chances of remission. Following both surgery and chemotherapy, the most successful individual cases were accompanied by the recovery of genotypes with active tumour suppression. If tumour suppression is not recovered, the treatment fails. Thus, if a large proportion of cells retain functional tumour suppressors at the time of therapeutic intervention, it is possible to recover less aggressive genotypes through treatment, leading to a better prognosis. Conversely, if tumour suppressor function is compromised prior to treatment in the bulk of the population, e.g. through field cancerization, the intervention can lead to an evolutionary bottleneck that selects for the rapid expansion of the most malignant cells in the population. Thus, while the speed of relapse in aggressive cases is dominated by the action of oncogenes that drive proliferation, a sustained recovery following treatment depends largely on tumour suppression.

These results suggest that targeting chromosomally unstable cells may be an important part of future cancer therapies. It has been suggested that chromosomal instability may also play a role later in generating the genetic diversity required for cancer cells to survive the trials of invasion and metastasis, something that we have not explored here. Only through the tracing of clear evolutionary pathways in real cancers will it be possible to understand the different roles that these complex mutations have throughout the process of carcinogenesis and thus help us to develop better treatments. Future work will be needed to assess how scrambling of the genome may combine with mis-segregation events to drive the evolution of chromosomes that have specific complements of genes.

In sum, in exploring the evolutionary pathway of cancer clones in tumour development our model shows the interplay between aneuploidy and tumour therapies. Future research will need to build on such models to being them closer to reality; to study the role of aneuploidy on more advanced kinds of tumours, and to simulate other kinds of cancer treatments.

## Methods

### Agent-based Model Algorithm

All cells are ordered in a linked list, and each cell has a simulated genome composed of three kinds of genes. Each of the three genes code for corresponding actions at a cellular level, inspired by biological systems and known cancer genes [12]. The genes present and their functions, described below, are:

- Tumour Suppressors- Apoptosis Regulatory Genes
- Proto-oncogenes- Cell Division Regulatory Genes
- Genetic stability- Chromosome Segregation Regulatory Genes

The homeostatic constraints in the model were abstracted from real biological systems, where the overall goal of homeostasis is to maintain the tissue’s relative constant size and shape [36]. The homeostatic size of the tissue is established for each experiment through an allocated space parameter, where measurements for homeostasis are based on global cell count. Although this model is not spatially explicit, there is a degree of spatial structure in the model since new daughter cells are introduced in the linked list of cells spatially replacing the cell of origin after division.

Inspired by the processes in biological cellular behaviour through which homeostasis is maintained in organisms, the algorithm is as follows:

1. An initial population of 100 cells is created, each with diploid chromosomes. Each initial genome was equipped with 2 copies of each type of gene, grouped into chromosomes according to the gene distribution type A, B or C. The normal carrying capacity of the tissue is fixed at 200 cells.
2. For each time step, the total number of cells is measured and is not updated until the next time step.
3. If a measurement of the total number of cells is greater than the tissue’s carrying capacity, then the probability of cell death is calculated. The probability of death depends on the number of available copies of the apoptosis regulatory genes, 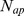, within each cell’s genome. The probability of apoptosis, 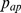, is determined by:

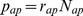
Where 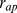 is a parameter for the rate of apoptosis. The cell is then killed with a probability of 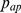.

1. If the cell has not died, it has a chance to divide. The probability of division depends on the number of available copies of the division regulatory genes, 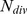, and a parameter that determines the rate of division, 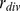. The probability that a cell divides, 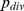, is:

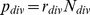

1. If dividing, a new cell is introduced in the linked list, spatially adjacent to the mother cell (Figure S1 A), and the probability of chromosome missegregation is calculated. If there is a chromosome missegregation event, one chromosome chosen at random is asymmetrically distributed during cell division leading to the creation of two aneuploid cells. Otherwise, the genome is duplicated and copied with fidelity, thus generating two identical daughter cells. The probability of chromosome missegregation, 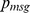, in the model is:

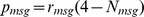
Where 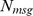 is the number copies of the chromosome segregation regulatory genes within the cell’s genome, and 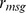 is a parameter for the rate of chromosome missegregation.

The update rules are applied synchronously to all the cells in each time step. Also, it is important to note that the probabilities are conditional (i.e one probability depends on the previous one, as described in the algorithm). For instance, the probability of chromosome missegregation seems to be the largest, but because it can only happen conditional to the probability of division, it is in reality low.

### Experiments

To investigate the properties and the dynamics of the system, and specifically the role that chromosome segregation regulatory genes have, four genome distributions were considered: Three reported here and a fourth distribution that considers every gene to be uncoupled as a control experiment. The parameter settings were determined through a series of preliminary experiments, in order to ensure that the behaviour of the system was both biologically plausible and computationally feasible. Simulations were carried out with the following initial parameters:

- Initial population: 100 cells
- Homeostatic size of the tissue: 200 cells
- Simulation end time: when reaching 7000 cells or reaching 300 time steps

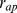
 *= 0.045, 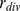 = 0.045, 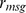 = 0.02*

### Treatments

Scenario i: Surgery: Once the size of the population had reached 1000 cells, a segment of the linked list that contains 900 cells is selected and deleted, leaving a residual segment of 100 (Figure S1 B). Scenario ii: Chemotherapy: An algorithm kills all the cells that attempt cell division in the nine consecutive time steps after the list has reached 1000 cells (Figure S1 C). Scenario iii: Combination Therapy. Surgery is implemented, followed by nine rounds of chemotherapy.

There is an important difference between the two treatments. When carrying out a surgical procedure, the cancer is removed along with some surrounding tissue at the tumour margin whose extent depends on the location and the extent of tumour invasion [29]. To capture these aspects of the treatment, surgery in the model was implemented as the removal of 90% of the cells in the “cell list”. The proliferation algorithm is implemented so that cell division generates related neighbouring cells.

### Genotype Key

For the analysis of the simulations, the emergent genotypes were assessed. By quantifying the number of chromosomes that a cell has at a given time, a genotype state 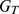 is defined as:
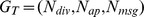



Where 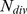, *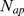 and* 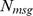 are the number of copies of *Cell Division Regulatory Genes, Apoptosis Regulatory Genes* and *Chromosome Segregation Regulatory Genes* respectively. The initial genotype consists of two functional copies of each chromosome: genotype state (2, 2, 2).

### RGB Key

Colours in the RGB model are defined by three components (Figure 3 A). Because of this, a three-dimensional volume is described by treating the component values as ordinary Cartesian coordinates in a Euclidean space. For the RGB model, this is represented by a cube using non-negative values within a 0–1 range, assigning black to the origin at the vertex (0, 0, 0), and with increasing intensity values running along the three axes up to white at the vertex (1, 1, 1), diagonally opposite black. An RGB triplet (red, green, blue) represents the three-dimensional coordinate of the point of the given colour within an RGB colour cube, or its faces or along its edges in a simplified version. This approach allows computations of the colour similarity of two given RGB colours by simply calculating the distance between them: the shorter the distance, the higher the similarity. We have taken advantage of this to describe the different genotypes that evolve in our system by normalizing the maximum observed Genotype State. We have assigned a colour to each of the abstracted genes: Red for division, green for death and blue for segregation. By comparing the similarity of the colours assigned to a given genotype, we are able to tell visually the proportions in which the genes are distributed, with intensity values corresponding to the number of genes: (0,0,0) being black, the initial genotype (2, 2, 2) being dark grey and the maximum observed genotype (5, 5, 5) being white.

### Statistical Test

For the statistical tests we used an unpaired t-test to determine if the means of the results in our two sets of experiments (Configuration B and C) are significantly different in key aspects of simulated treatments.

Our null hypothesis is that the observed response of the two configurations to treatments is due to chance. The alternative hypothesis is that the observed response to treatments depends on the configuration. For these tests, we have assumed a two-tailed distribution and equal variance.

## Supporting Information

Figure S1 **Actions within the linked lists of cells.** **A.** When dividing, a new cell is introduced in the linked list of cells, spatially adjacent to the mother cell. **B.** During surgery, a segment of the linked list that contains 900 cells is selected and deleted, leaving a residual segment of 100 cells. **C.** During chemotherapy, all the cells that attempt cell division in the nine consecutive time steps after the list has reached 1000 cells are deleted.
(TIFF)
