# Supplementary figures and images for: The Role of Chromosome Missegregation in Cancer Development: A Theoretical Approach Using Agent-Based Modelling
Source: PLoS One. 2013 Aug 26;8(8):e72206. doi: 10.1371/journal.pone.0072206 (PMC3753339; doi:10.1371/journal.pone.0072206)

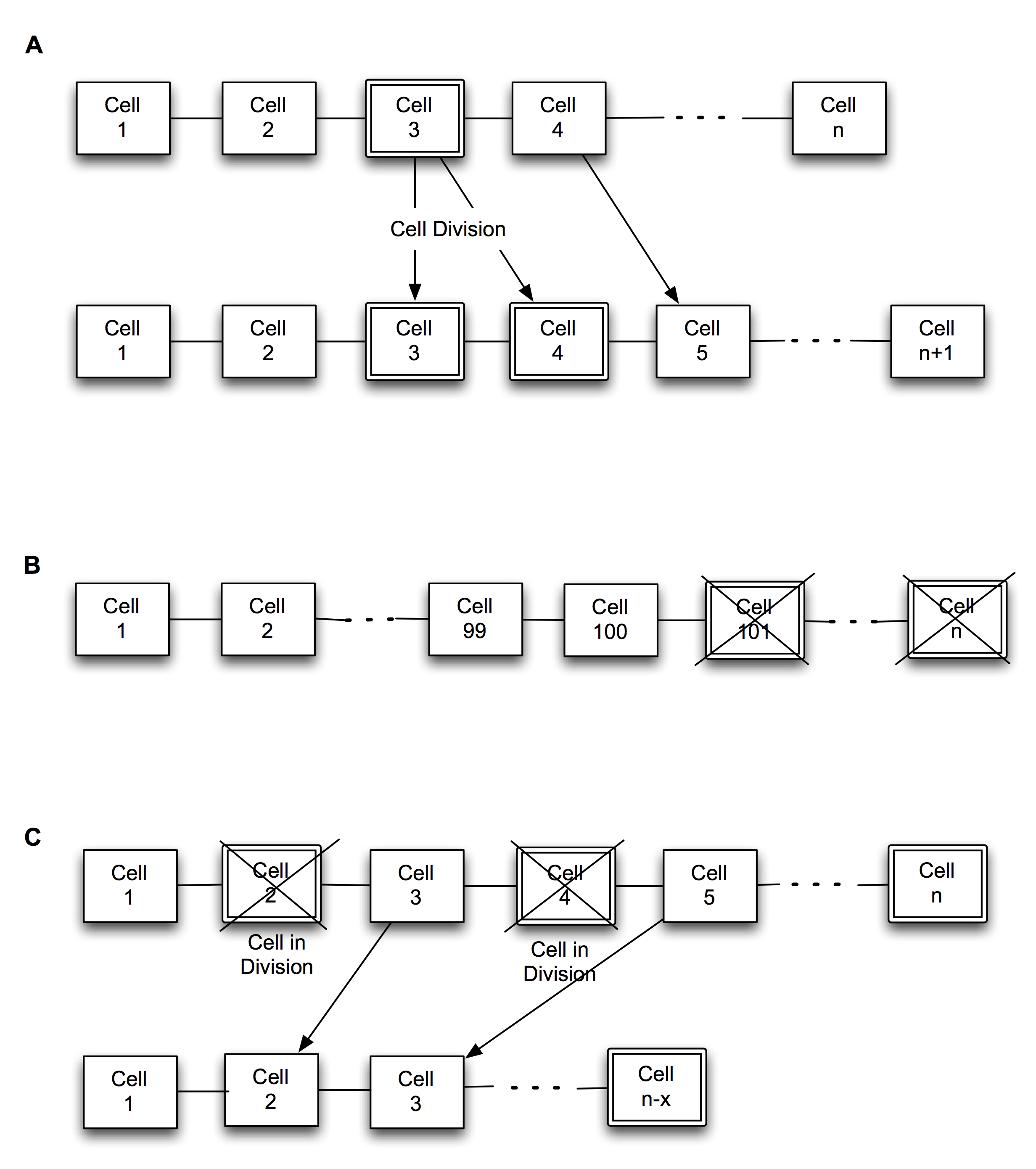

Supplement: Figure S1 — Actions within the linked lists of cells. A. When dividing, a new cell is introduced in the linked list of cells, spatially adjacent to the mother cell. B. During surgery, a segment of the linked list that contains 900 cells is selected and deleted, leaving a residual segment of 100 cells. C. During chemotherapy, all the cells that attempt cell division in the nine consecutive time steps after the list has reached 1000 cells are deleted. (TIFF) [file pone.0072206.s001.tif]
